# Antiplatelet/Anticoagulant Agents and Chronic Subdural Hematoma in the Elderly

**DOI:** 10.1371/journal.pone.0068732

**Published:** 2013-07-12

**Authors:** Pasquale De Bonis, Gianluca Trevisi, Chiara de Waure, Antonella Sferrazza, Massimo Volpe, Angelo Pompucci, Carmelo Anile, Annunziato Mangiola

**Affiliations:** 1 Institute of Neurosurgery, Catholic University School of Medicine, Rome, Italy; 2 Institute of Hygiene, Catholic University School of Medicine, Rome, Italy; 3 Health Department, Catholic University School of Medicine, Rome, Italy; Maastricht University Medical Center, The Netherlands

## Abstract

**Background and Purpose:**

In the last decade there has been an increasing use of antiplatelet/anticoagulant agents in the elderly. The aim of the study was to evaluate the association between exposure to anticoagulant/antiplatelet therapy and chronic subdural haematoma-CSDH.

**Methods:**

Single institution case-control study involving 138786 patients older than 60 years who visited our academic tertiary care Emergency Department from January 1st 2001 to December 31st 2010. 345 patients with CSDH (cases) were identified by review of ICD-9 codes 432.1 and 852.2x. Case and controls were matched with a 1∶3 ratio for gender, age (±5 years), year of admission and recent trauma. A conditional logistic model was built. A stratified analysis was performed with respect to the presence (842 patients) or absence (536 patients) of recent trauma.

**Results:**

There were 345 cases and 1035 controls. Both anticoagulant and antiplatelet agents were associated with an increased risk of CSDH with an OR of 2.46 (CI 95% 1.66–3.64) and 1.42 (CI 95% 1.07–1.89), respectively. OR was 2.70 (CI 95% 1.75–4.15), 1.90 (CI 95% 1.13–3.20), and 1.37(CI 95% 0.99–1.90) for patients receiving oral anticoagulants, ADP-antagonists, or Cox-inhibitors, respectively. History of recent trauma was an effect modifier of the association between anticoagulants and CSDH, with an OR 1.71 (CI 95% 0.99–2.96) for patients with history of trauma and 4.30 (CI 95% 2.23–8.32) for patients without history of trauma.

**Conclusions:**

Anticoagulant and antiplatelet therapy have a significant association with an increased risk of CSDH. This association, for patients under anticoagulant therapy, appears even stronger in those patients who develop a CSDH in the absence of a recent trauma.

## Introduction

Chronic subdural haematoma (CSDH) is predominantly a disease of the elderly (average age of onset 63 yrs). It usually follows a minor trauma and symptoms usually develop gradually over one to six weeks. However, a history of trauma is absent in up to half the cases. [1] Alcohol abuse, seizures, CSF shunts, coagulopathies including therapeutic anticoagulant, have traditionally been considered as other risk factors. [2] In the last decade there has been an increasing use of antiplatelet and anticoagulation therapy among adult patients, especially in the elderly. [3] This has also been encouraged by an increasing number of studies showing the clinical and economical advantage of aspirin assumption both for primary and secondary prevention of cardiovascular disease (CVD), as well as for cancer prevention [4], [5], [6], [7], [8], [9].

Bleedings are well known risks of both antiplatelet and anticoagulant therapy and both therapies have historically been considered as risk factors for CSDH. [10], [11], [12], [13], [14] Some Authors also reported an increased tendency to bilateral CSDH in patients under anticoagulant or antiplatelet therapy. [15] Moreover, according to a recent study, both these therapies may affect the quality of life after the surgical treatment for CSDH [16].

However, there is a lack of epidemiologic studies analysing the relationship between antiplatelet/anticoagulant therapy and the development of a CSDH. Therefore, the aim of this case-control study was to determine whether patients with antiplatelet/anticoagulant therapy were more likely to develop a CSDH than patients without antiplatelet/anticoagulant therapy.

## Materials and Methods

### Ethics Statement

The Catholic University Ethics Committee approved the present study. Written consent was given by the patients for their information to be stored in the hospital database and used for research. This study was performed following the principles outlined in the Declaration of Helsinki. The STROBE guidelines were followed for the preparation of this manuscript.

### Study Population

A case control study was carried out in order to investigate the association between anticoagulant/antiplatelet therapy and CSDH in patients older than 60 years. Ours is an academic tertiary referral center serving a population of 2 million people for emergencies.

Cases were identified by review of International Classification of Diseases-9 (ICD-9) codes 432.1 and 852.2x at Catholic University School of Medicine Emergency Department patients electronic database from January 1^st^ 2001 to December 31^st^ 2010. A total of 402 patients older than 60 yrs affected by both an acute or chronic subdural hemorrhage were initially retrieved. Medical records and imaging findings were then reviewed and only patients with chronic subdural hematoma were considered. Three hundred forty-five consecutive patients with CSDH older than 60 yrs were identified. Controls were selected among 138786 patients older than 60 yrs of age who visited the Emergency Department during the same years with a 3∶1 ratio with respect to cases. Case and controls were matched for gender, age (±5 years), year of admission and history of previous recent trauma (i.e. up to two months before hospital admission).

### Statistical Analysis

A descriptive statistic analysis was carried out by means of frequencies and mean and standard deviation (SD) for qualitative and quantitative variables respectively. A multivariable logistic model was built in order to evaluate the association between exposure to anticoagulant, antiplatelet and anticoagulant/antiplatelet therapy and CSDH; the analysis was adjusted by age, gender and previous trauma. Results have been reported as Odds Ratios (OR) and 95% Confidence Intervals (95% CI). In order to investigate the potential for effect modification, an interaction term was included in the model with respect to trauma and anticoagulant/antiplatelet therapy. The analysis was carried out using STATA 9.0.

## Results

The whole sample was represented by 345 cases and 1035 controls. The mean age of the sample was 77 years (SD: 8.3) and the majority (963; 69.8%) was represented by males. Among the 1380 total patients, 370 (23%) were taking an antiplatelet therapy while 118 (8.6%) were under an anticoagulant one. Among the 317 patients assuming antiplatelet therapy, 225 (71%) were taking COX inhibitors, 69 (21.8%) ADP antagonists and 18 (5.7%) were under dual antiplatelet therapy; for 5 (1.6%) patients, the information was missing. Among the 118 patients taking anticoagulants, 95 (80.5%) were under oral anticoagulant therapy (OAT), 22 (18.6%) under heparin and 1 (0.8%) was under unknown therapy. Study population characteristics stratified by the status are shown in Table 1.

**Table 1:** Study population characteristics stratified by the status.

		Cases (345)	Controls (1035)
**Age** *		77.2 (8.4)	77.2 (8.3)
**Gender**	Females	104 (30.1%)	313 (30.2%)
	Males	241 (69.9%)	722 (69.8%)
**Recent hystory of trauma**	No	134 (38.8%)	404 (39%)
	Yes	211 (61.2%)	631 (61%)
**Anticoagulant therapy (1379)**	No	296 (85.8%)	965 (93.3%)
	OAT	41 (11.9%)	54 (5.2%)
	Heparin	7 (2%)	15 (1.5%)
	Unknown	1 (0.3%)	–
**Antiplatelet therapy (1379)**	No	253 (73.3%)	809 (78.2%)
	Cox inhibitors	63 (18.3%)	162 (15.7%)
	ADP antagonists	24 (7%)	45 (4.3%)
	Dual antiplatelet	5 (1.4%)	13 (1.3%)
	Unknown	–	5 (0.5%)

* Mean (SD). OAT: oral anticoagulant therapy.

After dichotomizing the variables “antiplatelet therapy” and “anticoagulant therapy”, the assumption of either anticoagulant or antiplatelet therapy was shown to be associated with an increased risk of CSDH (Tables 2 and 3). Ten patients were taking both anticoagulant and antiplatelet therapy: their risk was shown higher (Table 3).

**Table 2:** Results from regression model.

	Cases (345)	Controls (1034)	OR (95% CI)∧
**Antiplatelet therapy**			**1.42 (1.07**–**1.89)**
Yes	92	225	
No	253	809	
**Anticoagulant therapy**			**2.46 (1.66**–**3.64)**
Yes	49	69	
No	296	695	

∧ adjusted for age, gender and previous trauma.

**Table 3:** Results from regression model.

	Cases (345)	Controls (1034)	OR (95% CI)∧
**No therapy**	209	745	**1**
**Antiplatelet therapy**	87	220	**1.42 (1.06**–**1.90)**
**Anticoagulant therapy**	44	64	**2.45 (1.62**–**3.71)**
**Antiplatelet/Anticoagulant therapy**	5	5	**3.61 (1.03**–**12.60)**

∧ adjusted for age, gender and previous trauma.

Excluding patients who were under unknown anticoagulant/antiplatelet therapy (n = 5 controls), we observed that the risk for CSDH development was significantly increased for patients receiving OAT (OR 2.70; 95% CI 1.75–4.15) or ADP antagonists (OR 1.90; 95% CI 1.13–3.20) (Table 4). Patients receiving Cox inhibitors had an increased -but not significant- risk of developing a CSDH (OR 1.37; CI 95% 0.99–1.90). The risk for CSDH was shown to be slightly but not significantly increased also for patients under dual antiplatelet therapy in comparison to patients assuming only one drug (OR 1.40; 95% CI 0.49–3.98). Finally, history of recent trauma seemed to play a role as potential effect modifier of the association between anticoagulant therapy and subdural hematoma; results stratified by trauma are shown in Table 5.

**Table 4:** Results from regression model – effect of drug classes.

		Cases	Controls	OR (95% CI)^∧^
**Antiplatelet therapy (1374)**	No therapy	253	809	1
	Cox inhibitors	63	162	1.37 (0.99–1.90)
	ADP antagonists	24	45	**1.90 (1.13**–**3.20)**
	Dual antiplatelet	5	13	1.40 (0.49–3.98)
**Anticoagulant therapy (1378)**	No therapy	296	965	1
	OAT	41	54	**2.70 (1.75**–**4.15)**
	Heparin	7	15	1.51 (0.61–3.75)

OAT: oral anticoagulant therapy; ^∧^adjusted for age, gender and previous trauma.

**Table 5:** Results from conditional regression model stratified by history of recent trauma.

	History of recent trauma (842)	No history of recent trauma (536)
	OR (95% CI)	OR (95% CI)
**Antiplatelet therapy**	1.35 (0.93–1.95)	1.54 (0.95–2.49)
**Anticoagulant therapy**	1.71 (0.99–2.96)	**4.30 (2.23**–**8.32)**
**Antiplatelet/Anticoagulant therapy**	3.51 (0.70–17.67)	3.83 (0.53–27.70)

## Discussion

Antiplatelet and anticoagulant therapy have traditionally been cited among risk factors for the development of CSDH. [10], [11], [12], [13], [14]. However, this statement is mainly based on individual case-series. In this case-control study we investigated the association between antiplatelet and anticoagulant therapy and CSDH.

Among the 345 cases, 92 patients (26.7% ) were taking antiplatelet drugs and 49 patients (14.2% ) were under anticoagulant therapy. In the control population, the percentage of patients taking antiplatelet and anticoagulant therapy was 21.8% and 6.7%, respectively. In both populations COX inhibitors and Vitamin K antagonists (Oral Anticoagulant Therapy) were the most frequently used drugs.

In a recent systematic review and meta-analysis of adverse events of low-dose aspirin and clopidogrel in randomized controlled trials, Mc Quaid and Laine provided evidence that low-dose aspirin was associated with an increased risk of intracranial hemorrhage (without specifying which type of intracranial hemorrhage) and major bleedings. [17] The authors also reported no difference between 75–162.5 mg/day and >162.5–325 mg/day aspirin. [17] A well described case-control study, instead, showed that the incidence of anticoagulant-associated intracerebral hemorrhage in the US population quintupled during the 90s and the majority of that change was explained by increasing warfarin use [3].

Our results confirm that anticoagulant and antiplatelet therapy have a significant association with an increased risk of CSDH, with an Odds Ratio of 2.5 (95% CI 1.68–3.71) and 1.42 (95% CI 1.07–1.88), respectively. The more significatively associated drug classes were Vitamin K antagonists among anticoagulants, OR(95% CI) 2.76 (1.78–4.27), and ADP antagonists among antiplatelets, OR(95% CI) 1.92 (1.14–3.24). Dual antiplatelet therapy showed a slight but not significant increased risk of CSDH, OR(95% CI) 1.14 (0.40–3.23). These data can be explained by the relatively low number of patients under dual antiplatelet therapy.

A history of trauma (usually a mild trauma which occurred between 2 weeks and 2 months before the symptoms onset) was referred by 211 out of 345 patients (61.16%) with CSDH. Among these patients, 59 (28%) were on antiplatelet therapy and 27 (12.8%) were on anticoagulant therapy. Among the remaining 134 CSDH patients with no referred history of recent trauma, 68 (50.75%) were taking antiplatelets and 24 (17.9%) were on anticoagulant therapy. Stratifying the analysis by trauma, both therapies showed an association with CSDH in the two categories: trauma and no trauma patients (Table 4). However a history of previous trauma seemed to play a role as potential effect modifier of the association between anticoagulant therapy and subdural hematoma. Interestingly, the association seemed to be stronger for patients with no history of recent trauma.

Obviously, our study has a number of limitations. The control population was selected among patients visited for other pathologies at our Emergency Department, assuming that the diffusion of the use of the analyzed drugs was representative of that of the general population of the same age. Notwithstanding confounding, a constraint in explanatory observational studies, may be possible, it should be observed that few factors are known to increase the risk for a chronic subdural hematoma. Major risk factors for bleeding in chronic subdural hematoma, which is the study outcome, are represented by age and trauma, that have been taken into account in matching process. Among other risk factors, alcohol, epilepsy, coagulopathies and CSF shunts may be listed. [2] All these factors were very uncommon in our cases, indeed they were not considered in the analysis.

Since the accuracy of a positive history of recent and often minor trauma depends from the patient’s collaboration and cognitive state or from patient/relatives knowledge of the previous history, the event might have been underestimated.

Another possible limitation could be that the use of the investigated drugs might have been omitted in the medical records of those patients who referred to A&E for other reasons. However, the Emergency Department electronic records include a detailed description of home therapy: data about drugs intake, as well as family and medical history, were collected by a dedicated physician at the Emergency Department through a standardized form. For this reason, even though misclassification was present, it would be non differential with respect to cases and controls.

We did not take into account the length of the chronic medical treatment. Moreover, we did not compare the drug dosage nor the INR values at presentation in the case of anticoagulants. However, drug dosage in the case of aspirin (within the recommended therapeutic range of 75–325mg/daily) has been demonstrated not to influence the complication rate. [17] Moreover, INR values at presentation could not reflect the INR values in the weeks before the admission (when traumatic event was supposed to happen).

This case-control study confirms that both anticoagulant and antiplatelet therapy have a significant association with an increased risk of CSDH. This association appears even stronger in those patients who develop a CSDH in the absence of a recent trauma. Since the exposure to antiplatelet and anticoagulant drugs increases the risk of developing a CSDH, as well as the risk of reoperation and of a lower quality of life after surgery, the indication to these therapies should strictly follow the current evidences in order to avoid a dangerous undue risk-benefit imbalance [16].
